# Evaluation of image quality between hybrid iterative reconstruction and deep learning reconstruction in low dose abdominopelvic CT in low body mass index individuals

**DOI:** 10.1038/s41598-026-52554-z

**Published:** 2026-05-08

**Authors:** C. Abraham Jacob, Nitika C. Panakkal, Shailesh Nayak, Rajagopal Kadavigere, Thejas M. Shivakumar, Jyoti Garg, Shivanath Shanbhag, Suresh Sukumar

**Affiliations:** 1 Department of Medical Imaging Technology, Manipal College of Health Professions, Manipal Academy of Higher Education, Manipal, India; 2 Department of Medical Imaging Technology, Manipal College of Health Professions, Manipal Academy of Higher Education, Manipal, Karnataka India; 3 Radio-Diagnosis & Imaging, Department of Radiodiagnosis & Medical Imaging, Kasturba Medical College, Manipal Academy of Higher Education, Manipal, Karnataka India

**Keywords:** Deep learning reconstruction, Low BMI, Image quality, Contrast to noise ratio, Signal to noise ratio, Image noise, Anatomy, Diseases, Health care, Medical research

## Abstract

The study aimed to compare image quality between hybrid iterative reconstruction and deep learning image reconstruction (DLIR) in low dose contrast enhanced CT (CECT) abdomen and pelvis scans in low body mass index (BMI) individuals, to determine whether DLIR provides superior image quality at a given dose. Fifty patients with BMI < 18.5 kg/m^2^ referred for CECT abdomen and pelvis were scanned on a 128-slice Philips CT scanner at 80 kVp. Images were reconstructed using iDose4 (level 4) and DLIR (precise image (PI), standard strength). Images were then analyzed quantitatively to estimate signal-to-noise ratio (SNR), contrast to noise ratio (CNR) and image noise by placing region of interest in aorta, liver, spleen and portal vein across the non-contrast, arterial and portal venous series. Qualitative analysis was performed by two radiologists using a 5-point Likert scale based on five image quality criteria and artifacts were assessed separately using 4-pont Likert scale. Paired t test/ Wilcoxon signed rank test was used to compare image quality parameters between both reconstruction algorithms. The mean attenuation (HU) did not significantly differ between the two reconstruction techniques for all the organs in the non-contrast, arterial and portal venous series. A significant increase in the SNR _liver_ was noted using precise image-standard across the non-contrast (idose4: 6.3 ± 1.7 vs PI: 6.6 ± 1.7, *p* < 0.001), arterial (idose4: 7.2 ± 1.9 vs PI: 7.7 ± 1.9, *p* < 0.001) and portal venous (idose4: 12.9 ± 2.41 vs PI: 13.7 ± 2.43, *p* < 0.001) phases. Similarly, CNR _liver_ significantly increased across the non-contrast [idose4: 0.09(− 0.4,0.6) vs PI: 0.4(− 0.3, 1), *p* < 0.001] and portal venous [idose4: 6 (4,7.17) vs PI: 6.47 (4.49, 7.62) *p* < 0.001] phases. Image noise was significantly lower with PI compared to iDose4 in the non- contrast (iDose4: 7.87 ± 0.97 vs PI: 7.59 ± 0.93, p value = 0.001), arterial (iDose4: 10.4 ± 1.42 vs PI: 9.83 ± 1.29, *p* < 0.001) and portal venous (idose4: 10.1 (9.6, 11.1) vs PI: 9.4 (8.9, 10.2), *p* < 0.001) phases. In qualitative analysis, Interobserver agreement between the two radiologists demonstrated good to excellent agreement (ICC range: 0.70–0.96). All the images were graded 3, 4 or 5, indicating good to excellent image quality with no artifacts. DLIR (Precise image-standard) offers a promising approach for improving image quality in low-dose abdominal and pelvic CT scans, particularly in individuals with a low BMI.

**Supplementary Information:**

The online version contains supplementary material available at 10.1038/s41598-026-52554-z.

## Introduction

Computed tomography (CT) has evolved over the years with faster acquisition speeds and technological advancements. Nevertheless, these advancements have resulted in higher radiation doses^1^. CT examinations of the chest, abdomen, and pelvis may involve relatively higher effective doses depending on the clinical protocol. On the other hand, contrast-enhanced CT (CECT) of the abdomen and pelvis serves as a prominent diagnostic tool and plays a crucial role in diagnosing a wide range of diseases^2^. However, these scans include multiple phases, making radiation exposure reduction one of the challenges in CECT abdomen and pelvis scans^3^. The radiation dose can be reduced using various dose reduction strategies including adjustment of exposure parameters such as tube potential, tube current, gantry rotation time, table speed, pitch and so on^4^. One of the major challenges associated with radiation dose reduction is increased image noise^5^. Reducing radiation dose in CT without compromising image quality can now be achieved by recent innovations in image reconstruction techniques. For many years, various iterative reconstruction (IR) approaches have been used to remove noise algorithmically and improve image quality at lower doses^6^. However, the IR technique raises concerns regarding image texture, as increasing IR strength can result in “blotchy” and “plastic” appearance due to its non-linear noise suppressing technique. This variability is further influenced by the types of iterative reconstruction algorithms used by different CT manufacturers and by the data processing domain in which they operate^7–9^. Some studies also report a loss of low contrast resolution at lower doses using IR techniques, which can influence the visualization of lesions in abdominal organs^10,11^. With the emergence of deep learning image reconstruction (DLIR) techniques, the challenges of image noise, artifacts and reduced image resolution have been addressed^12^. There are multiple approaches for devising deep learning techniques, one of which includes a convolutional neural network (CNN) trained on low-dose noisy images to generate images with a quality of routine standard-dose acquisition with lower noise and maintained resolution^13^. Clinical studies performed for abdominal regions have demonstrated the superior performance of DLIR compared with that of IR^13–15^. Very few studies have investigated the influence of body size on image quality using DLIR techniques at lower kVp. Wang et al. reported superior performance of DLIR in both image quality and lesion conspicuity in individuals with a wide range of body size indices at lower kVp^12^. However, the study was based on an anthropometrically different population compared with South Asians. South Asian individuals predominantly have a higher body fat percentage with lower lean body mass^16,17^. These differences in body composition may influence the attenuation characteristics and contrast perception. Moreover, in low BMI individuals, the use of lower tube voltage may be associated with reduced photon flux, potentially leading to increased image noise and affecting the overall image quality. Therefore, the present study aimed to compare image quality between hybrid iterative reconstruction and DLIR for low-dose CECT of the abdomen and pelvis in individuals with a low BMI.

## Materials and methods

A prospective study was carried out after approval from institutional ethical committee of (IEC38/2024). The research was carried out in accordance with relevant guidelines and regulations. The sample size was estimated a priori for a paired t test, assuming a two tailed significance level of 0.05, 80% power and a moderate effect size 0.45. Informed consent was obtained from 50 patients aged 18 years or older who underwent CECT of the abdomen and pelvis. Patients were consecutively recruited between 2024 and 2025. Demographic details such as age, height, weight were noted. Body mass index (BMI) was calculated as weight in kilograms divided by height in meters squared (kg/m^2^). A BMI of < 18.5 kg/m^2^ was used to classify individuals as having low BMI based on world health Organization (WHO) criteria for Asian population^18^. Patients with hepatic conditions that may alter liver attenuation such as fatty liver, cirrhosis or hepatic cell carcinoma which may require a higher contrast dosage and hepatic space occupying lesions in whom ROI placement could not be achieved were excluded. All the patients were scanned in a Philips 128-slice Incisive CT scanner using 80 kVp with tube current modulation (reference range from 80 to 350 mA) and a dose right index (DRI) of 17 which constitutes the routinely employed protocol for low BMI individuals at our institution. The other scanning parameters included 1.20 (pitch), 0.50 s (rotation time), 64 × 0.625 (detector collimation) and 768 × 768 (matrix size). Scanning coverage was from the domes of the diaphragm up to the pubic symphysis. After the non-contrast scan, a fixed contrast volume of 60 mL of Omnipaque 350 and 40 mL of saline were injected at a flow rate of 3.5 mL/sec. Bolus tracking was performed with the region of interest placed in the abdominal aorta and triggering threshold of 150 HU. Following contrast agent administration, arterial and portal venous phase images were acquired at 8 s and 45 s, respectively. All images were reconstructed from the same raw data set using 4^th^ generation hybrid iterative reconstruction (iDose4) level 4, and precise image (PI), standard level, Philips. The iDose4 combines both filtered back projection and iterative reconstruction for reducing noise but is constrained over slower reconstruction times^19^. PI is a deep learning based reconstruction that utilizes a convolutional neural network to enhance image quality at faster speeds^20^. Although smoother levels (e.g., smooth and smoother) of PI were available, only the standard level was used as it represents the routine clinical setting in our institution and avoids bias related to excessive noise suppression. The reconstructed images were then analyzed quantitatively and qualitatively.

### Quantitative image analysis

**Fig. 1:**
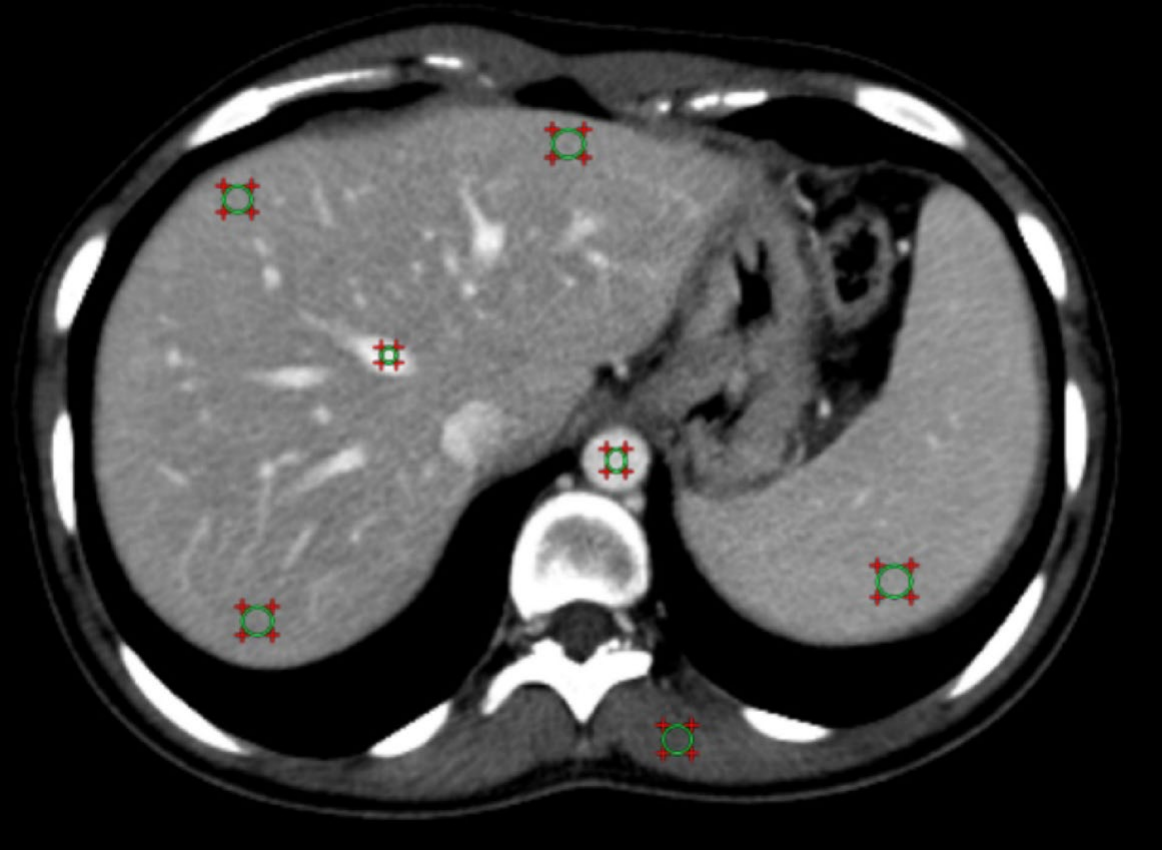
ROI placement for various organs and vessels.

All images were viewed with a DICOM viewer (Bee DICOM viewer, SinoUnion healthcare, Inc.), and regions of interest (ROIs) were delineated in various anatomical structures, mainly the liver, spleen, aorta, portal vein, and paraspinal muscle, in the plain, arterial, and portal venous phases. ROIs were placed at identical anatomical locations in both reconstructed images to ensure consistency in measurements. An ROI of size of 100 mm^2^ was used in organs, and 25 mm^2^ was used for the vessels (Fig. 1).

Within the liver, specific regions of interest (ROIs) were positioned in three distinct locations: the right anterior lobe, right posterior lobe, and left lateral segments. In the spleen, ROIs were placed in homogeneous parenchyma, avoiding vessels and artifacts. In the aorta, the ROI was positioned at the anatomical level corresponding to the celiac artery. In the portal vein, the ROI was positioned at the confluence level of the portal vein. Each calculated values were obtained by averaging measurements from two consecutive image slices. Image noise was estimated using the standard deviations of attenuation values measured within ROIs placed in abdominal organs. The signal-to-noise ratio (SNR), contrast-to-noise ratio (CNR) were subsequently computed using Eqs. 1 and 2^21,22^.

1 $$CNR = - \frac{CT\;number\;ROI\;organ - CT\;number\;ROI\;muscle}{{N\;\left( {SD\;of\;paraspinal\;muscle} \right)}}$$

Since paraspinal muscle was used as the reference tissue, CNR values may be negative when the attenuation of the target structure is lower than that of muscle (e.g., unenhanced aorta). These negative values reflect relative contrast differences and do not indicate reduced image quality. Statistical comparisons were performed using calculated CNR values without taking absolute values.

2 $$SNR = \frac{CT\;number\;ROI\;organ}{{SD\;organ}}$$

### Qualitative image analysis

Qualitative analysis was performed independently in a blinded manner by two radiologists, each with more than 5 years of experience. In case of disagreement, a third radiologist was consulted to reach a consensus. Window width and window level were allowed to be adjusted as needed by the reader according to routine clinical practice. Qualitative assessment was performed using a 5-point Likert scale based on the European guidelines on quality criteria^23^ for various criteria, including visualization/conspicuity, critical reproduction, and visualization of large vessels, with (1) indicating structures that cannot be identified/blurry, (2) is suboptimal, (3) is acceptable, (4) better than acceptable, and (5) indicating excellent visualization. For image contrast and image noise, (1) indicated poor image contrast/unacceptable image noise, (2) for suboptimal image contrast/above average image noise, (3) for acceptable image contrast/average image noise, (4) for above average image contrast/less than average noise, and (5) for excellent image contrast/minimal image noise. As the Likert scale used did not assess image artifacts, these were evaluated separately using a 4-point Likert scale^24^. Here, (1) indicated artifacts affecting diagnostic information, (2) for major artifacts affecting the visualization of major structures but the diagnosis is still possible, (3) for minor artifacts not interfering with the diagnostic decision, and (4) indicating no artifacts.

### Statistical analysis

The data were analyzed with Jamovi statistics 2.6.22. Demographic and image quality parameters (SNR, CNR, and image noise) were summarized using descriptive statistics. Normality of the data was assessed using the Shapiro–Wilk test. Normally distributed variables were expressed in mean ± standard deviation while non- normally distributed data was expressed as median (interquartile range). To compare quantitative image quality between the two reconstruction techniques, a paired t-test was used for normally distributed data, and a Wilcoxon signed rank test was used for skewed distributed data. Wilcoxon signed rank was used to compare qualitative image quality. Interobserver agreement was assessed using the intraclass correlation coefficient (ICC) based on a two-way mixed-effects model with absolute agreement. A *p* value < 0.05 was considered statistically significant.

## Results

### Patient characteristics

Out of the 50 patients included in the study, 29 (58%) were males, and 21 (42%) were females. The mean age was found to be 46.8 ± 17 years with an average BMI of 16.9 ± 1.59 kg/m^2^. Radiation dose for the 80 kVp protocol were as follows: CTDI _vol_: 39.3 ± 7.6 mGy; Dose Length Product: 714 ± 114 mGy*cm; Effective Dose: 10.0 ± 1.7 mSv^29^.

### Quantitative image analysis

The mean CT number did not significantly differ between the two reconstruction techniques for all the organs in the non-contrast, arterial, and portal venous phases, as shown in Table 1.

**Table 1:** Mean/median CT number in iDose4 and the precise image.

Series	Organs	CT attenuation (HU)		
		iDose4	PI	p value
Plain	Aorta	39.3 ± 5	38.4 ± 5.3	0.08
	Liver	46.5 (41.9,53.9)	48.5 (43.8, 54.4)	0.05
	Spleen	49.1 ± 4.68	48.1 ± 3.69	0.09
Arterial	Aorta	500 ± 115	502 ± 116	0.10
	Liver	65.4 (61, 71.1)	64.8(60.2,71.9)	0.09
	Spleen	134 ± 37.6	135 ± 37	0.16
Portal venous	Aorta	186 (164, 201)	188 (165,201)	0.41
	Liver	118 ± 16.9	118 ± 17.1	0.72
	Spleen	126(116,138)	125 (117, 138)	0.97
	Portal Vein	186 ± 26.7	188 ± 30	0.30

Normally distributed data are presented as mean ± standard deviation and compared using a paired t-test; non-normally distributed data are presented as median (interquartile range) and compared using the Wilcoxon signed-rank test.

A significant increase in the SNR was observed with PI compared to iDose4 across various organs in the non-contrast, arterial and portal venous phases as shown in Table 2.

**Table 2:** Mean/median signal-to-noise ratio (SNR) in iDose4 and the precise image.

Series	Organs	iDose4	PI	*p* value
Plain	Aorta	4(3.2,4.5)	4.07 (3.62, 4.50)	0.16
	Liver	6.3 ± 1.7	6.6 ± 1.7	< 0.001*
	Spleen	6.2 ± 1.1	6.4 ± 1.1	0.01*
Arterial	Aorta	43.9(36.9,56.3)	44.7 (39.9,53)	0.42
	Liver	7.2 ± 1.9	7.7 ± 1.9	< 0.001*
	Spleen	12.3 ± 3.8	13.7 ± 4.5	< 0.001*
Portal venous	Aorta	16.9(14,20.3)	18.2(16.4,22)	< 0.001*
	Liver	12.9 ± 2.41	13.7 ± 2.43	< 0.001*
	Spleen	13.4 ± 3.30	14.4 ± 3.22	< 0.001*
	Portal Vein	17.2 ± 4	18.4 ± 4.6	< 0.001*

Normally distributed data are presented as mean ± standard deviation and compared using a paired t-test; non-normally distributed data are presented as median (interquartile range) and compared using the Wilcoxon signed-rank test.

CNR was also found to be higher with PI; however, the increase was not significant in the liver for arterial phase or for organs such as the aorta and spleen in the non-contrast phase (Table 3).

**Table 3:** Mean/median contrast-to-noise ratio (CNR) in iDose4 and the precise image.

Series	Organs	IDose4	Precise Image	p value
Plain	Aorta	− 0.932 (− 1.35, − 0.5)	− 0.95 (− 1.65, − 0.5)	0.92
	Liver	0.09(− 0.4,0.6)	0.4(− 0.3, 1)	0.00*
	Spleen	0.1 ± 0.8	0.2 ± 1	0.38
Arterial	Aorta	44.2(35.9, 54.2)	49.2(37.9, 57.3)	< 0.001*
	Liver	1.48(0.5, 2.4)	1.67(0.4, 2.4)	0.78
	Spleen	8.52 ± 4	9.19 ± 4.2	< 0.001*
Portal venous	Aorta	13.2(10.5, 15.5)	14(11.2, 16.3)	< 0.001*
	Liver	6 (4, 7.17)	6.47 (4.49, 7.62)	< 0.001*
	Spleen	6.39(5.4, 7.7)	6.94(5.9, 8)	< 0.001*
	Portal Vein	12.7 ± 3.39	13.9 ± 3.89	< 0.001*

Normally distributed data are presented as mean ± standard deviation and compared using a paired t-test; non-normally distributed data are presented as median (interquartile range) and compared using the Wilcoxon signed-rank test.

Image noise was significantly reduced when PI was used across the non-contrast (iDose4 7.87 ± 0.97 vs PI 7.59 ± 0.93; p value = 0.001), arterial (iDose4 10.4 ± 1.42 vs PI 9.83 ± 1.29; *p* value < 0.001) and portal venous phase (iDose4 10.1 (9.6,11.1) vs PI 9.4 (8.9,10.2); *p* value < 0.001).

### Qualitative image analysis

**Fig. 2:**
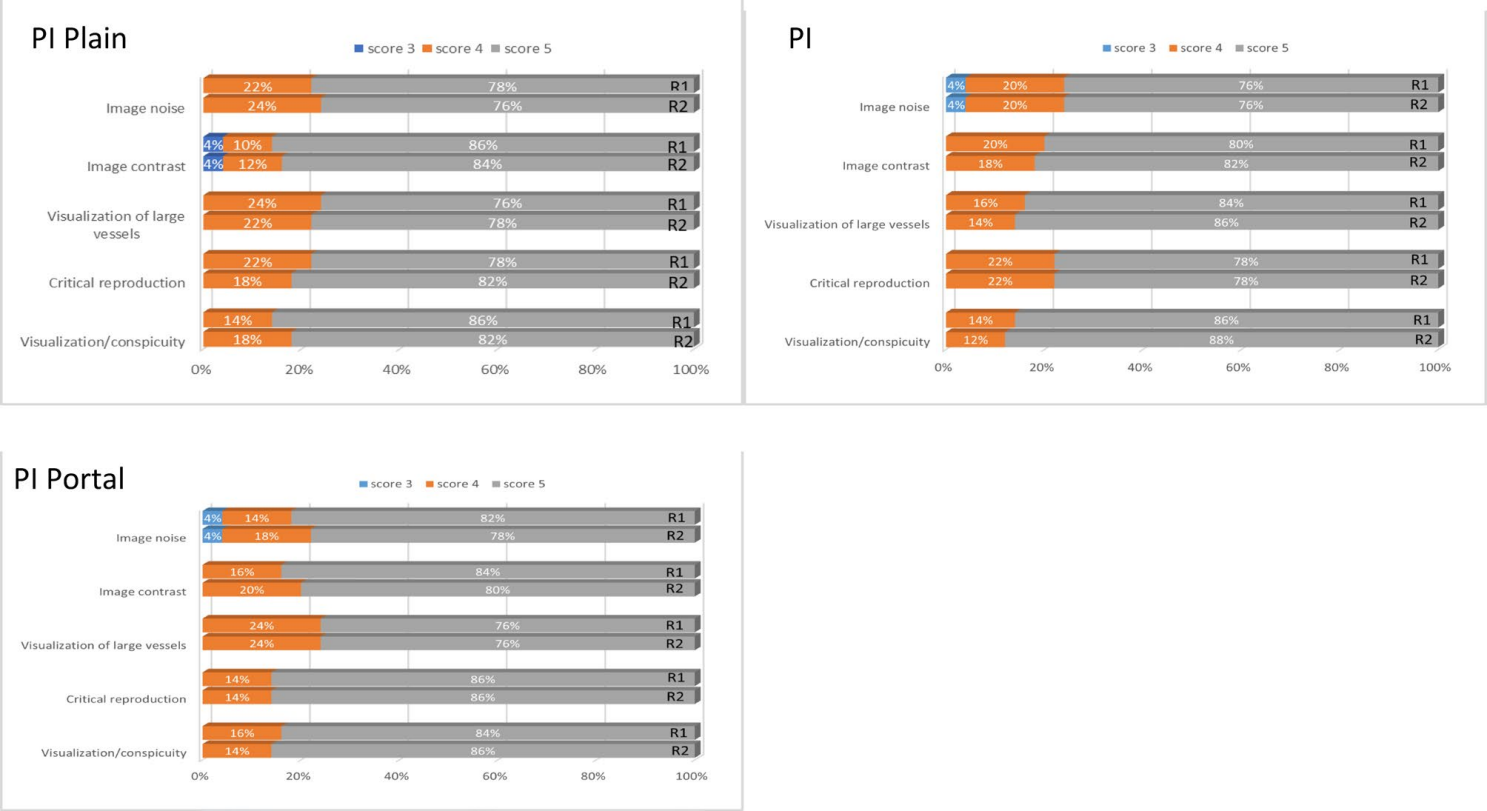
Percentage distribution of image quality scores assigned by two raters (R1, R2) for PI across plain, arterial and portal venous phase.

**Fig. 3:**
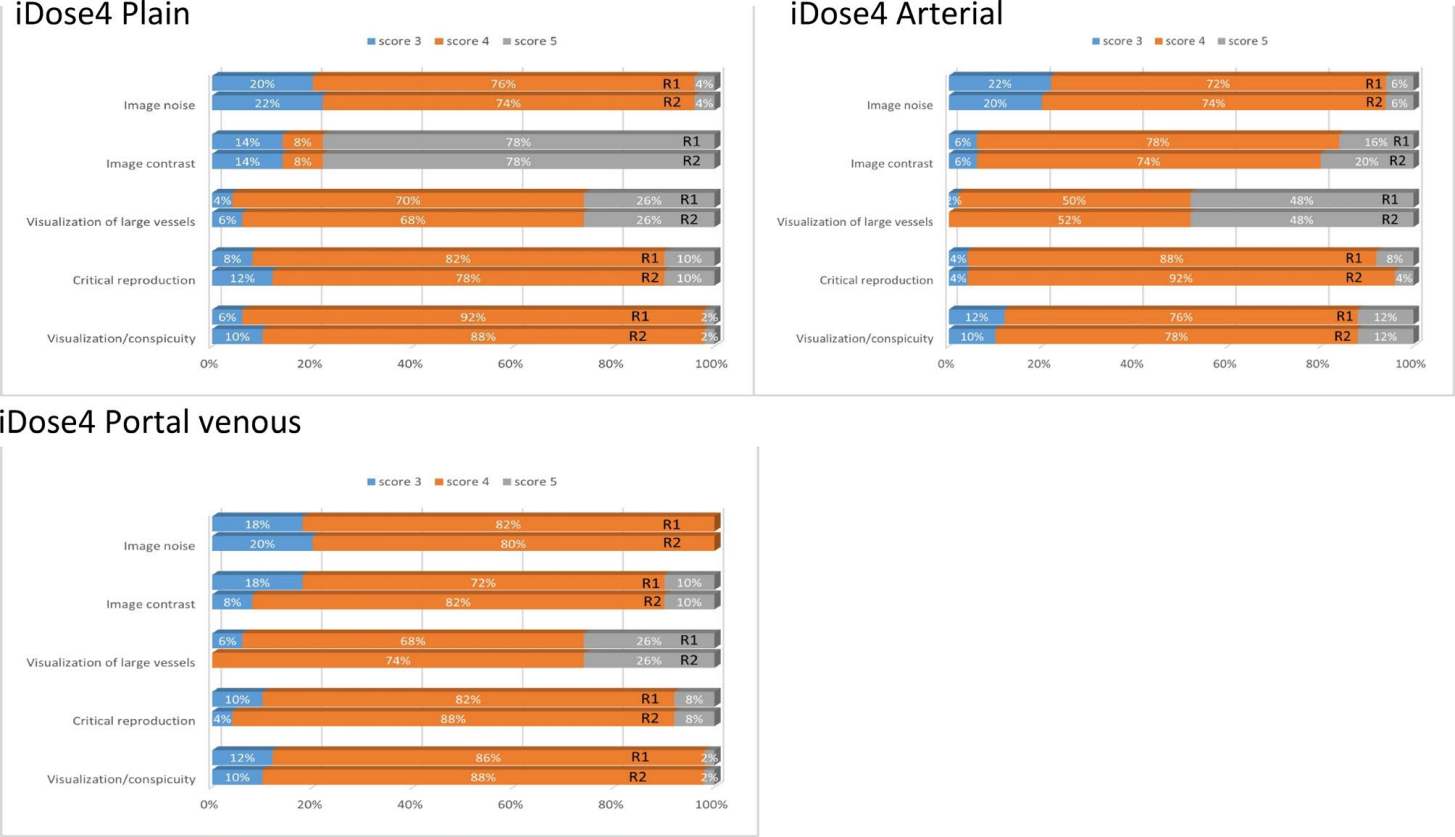
Percentage distribution of image quality scores assigned by two raters (R1, R2) for iDose4 images across plain, arterial and portal venous phase.

The intraclass correlation agreement between the readers ranged from 0.70 to 0.96, indicating good agreement. The lowest agreement (ICC = 0.703) was observed for image contrast criteria in the portal venous phase using iDose4 while all other criteria demonstrated higher levels of agreement. The ICC scores for different image quality criteria has been provided in the supplementary file. None of the images were scored 1 and 2 indicating poor quality in both reconstruction techniques. The percentage distribution of image quality scores for PI and iDose4 images across plain, arterial and portal venous phases are given in Figs. 2 and 3.

**Fig. 4:**
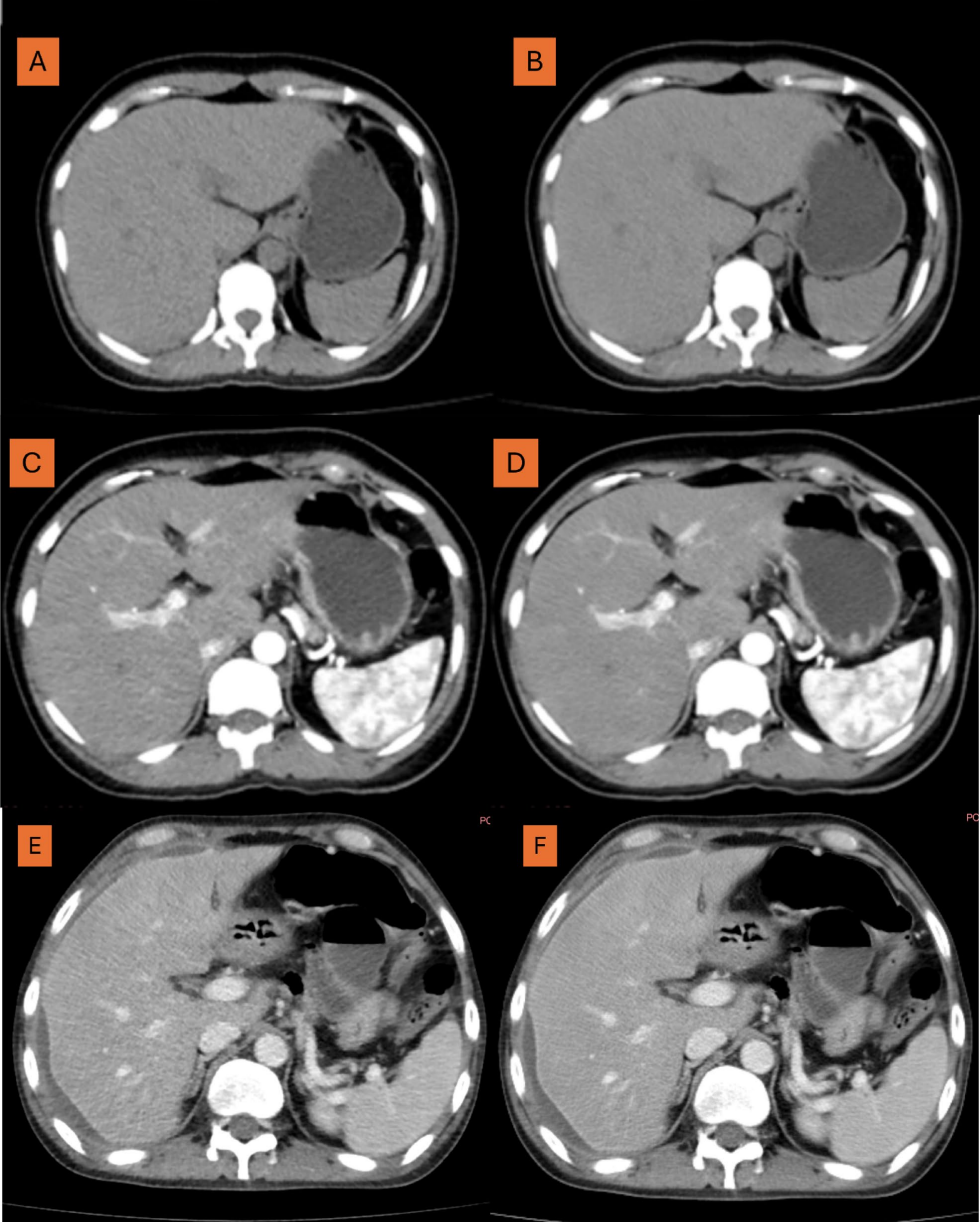
Non- contrast axial CT images of abdomen and pelvis in a 30- year-old male (BMI: 18.5 kg/m^2^). Image (**A**) reconstructed using iDose4 level 4 and image (**B**) reconstructed using PI (standard level). Images C and D representative of contrast arterial phase of 38 -year-old female (BMI: 17.3 kg/m^2^). Image (**C**) reconstructed using iDose4 level 4 and image (**D**) reconstructed using PI (standard level). Images E and F representative of contrast portal venous phase of 46 -year-old male (BMI: 17.7 kg/m^2^). Image (**E**) reconstructed using iDose4 level 4 and image (**F**) reconstructed using PI (standard level) Images are displayed with a window width of 350 and window level of 40.

With respect to image artifacts, no images were assigned the lowest score (1 and 2). Images graded as 4 constituted iDose4 [PL (73%), A (75%), PV (71%)] and PI [PL (81%), A (82%), PV (81%)]. Across all image quality criteria, PI demonstrated significantly better performance than iDose4 as depicted in Table 4. Figure 4 demonstrates image quality comparison for non-contrast and various contrast enhanced phases between iDose4 and PI.

**Table 4:** Comparison of qualitative image quality parameters between iDose4 and PI.

Qualitative parameters	Plain			Arterial			Portal venous		
	iDose4	PI	*p* value	iDose4	PI	*p* value	iDose4	PI	*p* value
Visualization/Conspicuity	4 (4, 4)	5 (5, 5)	< 0.001	4 (4, 4)	5 (5, 5)	< 0.001	4 (4, 4)	5 (5, 5)	< 0.001
Critical reproduction	4 (4, 4)	5 (5, 5)	< 0.001	4 (4, 4)	5 (5, 5)	< 0.001	4 (4, 4)	5 (5, 5)	< 0.001
Visualization of large vessels	4 (4, 4.75)	5 (5, 5)	< 0.001	4 (4, 5)	5 (5, 5)	< 0.001	4 (4, 4.75)	5 (5, 5)	< 0.001
Image contrast	4 (4, 4)	5 (5, 5)	< 0.001	4 (4, 4)	5 (5, 5)	< 0.001	4 (4, 4)	5 (5, 5)	< 0.001
Image noise	4 (4, 4)	5 (5, 5)	< 0.001	4 (4, 4)	5 (5, 5)	< 0.001	4 (4, 4)	5 (5, 5)	< 0.001
Artifacts	4 (3.13, 4)	4 (4, 4)	< 0.001	4(3.63, 4)	4 (4, 4)	< 0.001	4 (3.13, 4)	4 (4, 4)	< 0.001

Values are expressed as median (interquartile range, IQR). As the qualitative parameters are ordinal variables, non-parametric tests were used for statistical comparison.

## Discussion

Numerous studies have demonstrated the robust performance of DLIR in abdominal imaging^15,25–27^. While DLIR does not directly reduce the radiation dose, it enables greater dose reduction by improving image quality and thereby allowing lower-dose imaging protocols to be feasible. In a review by Ahmad et al., the use of DLIR reduced the radiation dose by 30–77%^28^. Although radiation dose was not assessed in the present study, the 80 kVp protocol employed here was previously validated, with a reported dose reduction of up to 70% in low BMI individuals for triple-phase CECT of the abdomen and pelvis in the prior work^29^. The present study was designed to evaluate the performance PI within a low kilovoltage (80 kVp) contrast-enhanced abdomen and pelvis protocol on all 3 phases, namely, non-contrast, arterial and portal venous phases, in low-BMI individuals. A significant improvement in image quality using PI (standard) was observed across most organs across all the phases. This improvement is likely attributable to reduced image noise, while the absence of significant differences in CT attenuation values between reconstruction algorithms indicates preservation of Hounsfield unit accuracy. Different studies comparing DLIR algorithms to IR algorithms have reported similar results^25,30,31^. Although the SNRs and CNRs for all organs improved using PI, the difference was not significant enough for the SNR_aorta_/CNR_aorta_ and CNR_spleen_ in the plain series. These results could be attributed to the effectiveness of the iDose4 algorithm at preserving edge sharpness^32^ or maybe attributed to the higher baseline signal due to homogenous blood. Similarly, no significant improvement in SNR_aorta_ or CNR_liver_ was observed in the arterial phase with PI which may be explained due to phase specific factors, including the inherently high baseline signal in the contrast enhanced aorta and the relatively limited CNR in the liver due to heterogenous arterial enhancement; additionally, the comparable standard deviation of the paraspinal muscle between reconstruction techniques may have further limited any observable improvement in CNR. Although previous studies have demonstrated significant improvements in image quality across organs using DLIR, variations in findings across studies may be attributed to differences in reconstruction algorithms and choice of reconstruction strength, both of which can influence image quality metrics. Moreover, all images in the present study were reconstructed using the PI standard level as opposed to the varying strength levels employed in other studies, which may partly account for the observed differences in noise reduction and image quality metrics as shown in Table 5.

**Table 5:** Reported image quality metrics in literature.

Studies	kVp	Reconstruction algorithm	Image noise	CNR_liver_
Present study	80	Precise image	9.4	6.4
Motonori A et al.^25^	120	AiCE	14.6	2
Hyo Jin et al.^26^	100	TrueFidelity	32.5	10.6
Tormund et al.^27^	120	TrueFidelity	9.3	–
Le Cao et al.^31^	120	TrueFidelity	14.7	2.48
Jensen et al.^33^	120	TrueFidelity	9.9	6.5
Wang et al.^12^	80	TrueFidelity	13.6	4.5

In a phantom study, Greffier et al. reported that noise levels were lower when smooth and smoother reconstruction levels were used than when iDose4 was used and that smooth levels were better at achieving adequate image quality at lower doses particularly the conspicuity of liver metastasis, when these settings were applied^34^. In addition, differences in body size may also affect image noise and related quantitative metrics. A study conducted by Wang et al.^12^ reported a significant improvement in image quality and hepatic lesion conspicuity when DLIR medium and high strengths were used across various BMI groups in the portal venous phase. The present study focused exclusively on patients with a low body mass index (BMI). Although both studies used 80 kVp for low-BMI individuals, the differences in image quality variables could be attributed to different BMI ranges. In contrast to Wang et al.’s study, which included individuals with a BMI < 23.9 kg/m2 in the low BMI category, the present study included individuals with a BMI of only < 18.5 kg/m2. These differences in patient body habitus, along with variations in vendor specific reconstruction techniques, slice thickness employed (1.25 mm to 5 mm) and imaging protocols, may contribute to the observed differences in image quality across studies (Table 5). There was also variability in how noise was estimated in studies reported in literature. The present study estimated image noise by averaging the SDs of all organs, namely, the aorta, liver, spleen and portal vein, as opposed to noise estimated from muscle^25,31^ and fat^12,15,26,33^. Combining standard deviations from multiple organs to estimate image noise has been reported in several studies^35–37^ and may help minimize bias associated with single organ measurements while improving the consistency and representativeness of data. Nevertheless, subjective image assessment demonstrated significantly higher scores for PI across all image quality criteria. The higher scores for image quality criteria such as critical reproduction and visualization of large vessels may enhance the identification of organ planes and lesion margin, thereby potentially increasing diagnostic confidence, particularly in low BMI individuals where reduced intra-abdominal fat limits natural contrast between structures. These findings are consistent with prior literature and support the robustness of DLIR across different clinical settings.

This study has some limitations. First, higher iDose4 levels were not evaluated as it represented the routine reconstruction protocol for low dose abdominal CT. Following the introduction of PI only standard settings were evaluated at higher settings (smooth and smoother levels), while offering greater noise reduction, may lead to over-smoothing and alteration of image texture. Future studies can investigate the impact of these higher smoothing levels on image texture and diagnostic performance. Moreover, comprehensive assessment of noise characteristics including noise power spectrum is important when evaluating non-linear reconstruction algorithms such as DLIR as it enables characterization of noise texture and spatial frequency distribution beyond simple noise magnitude. There is potential for ROI measurement bias, as quantitative analysis was not performed in a blinded manner, however this was mitigated by measuring multiple ROIs across consecutive slices and using the average attenuation. Furthermore, this was a single-center study and the use of vendor-specific reconstruction algorithms may limit the applicability of the results to other CT systems. The study included only individuals with a lower body mass index as this group allows the use of lower tube voltages such as 80 kVp resulting in substantial dose reduction and increased image noise. Furthermore, while the impact of DLIR on image quality has been well studied in normal BMI populations^12,20^, conducting studies on individuals with a higher BMI can provide a comprehensive evaluation of the DLIR algorithm and its potential to enhance image quality across a group that typically presents with greater noise challenges at lower doses. In addition, the present study focused on image quality assessment without direct evaluation of lesion detection performance; therefore, its impact on diagnostic accuracy remains uncertain.

## Conclusion

In this study of patients with low BMI, the DLIR image reconstruction algorithm demonstrated a significant reduction in image noise and improved signal to noise ratio and contrast to noise across the non-contrast, arterial and portal venous phases compared with hybrid iterative reconstruction. These findings were supported by qualitative image assessments. Overall, DLIR image reconstruction may offer a promising approach for improving image quality in low-dose abdominal and pelvic CT imaging in low BMI individuals.

## Supplementary Information

Below is the link to the electronic supplementary material.

Supplementary Material 1

## Data Availability

The data supporting the findings of this study are not publicly available due to patient privacy. Data are available upon reasonable request from the corresponding author, subject to institutional and ethical approvals.
