# Supplementary material for: Evaluation of image quality between hybrid iterative reconstruction and deep learning reconstruction in low dose abdominopelvic CT in low body mass index individuals
Source: Sci Rep. 2026 May 8;16:21176. doi: 10.1038/s41598-026-52554-z (PMC13342276; doi:10.1038/s41598-026-52554-z)
Supplement: Supplementary file 1 — Supplementary Material 1 [file 41598_2026_52554_MOESM1_ESM.docx]

**Table 1a:** ICC-based interobserver agreement for qualitative image quality parameters

| **Qualitative parameters** | **Plain** | | | |
| --- | --- | --- | --- | --- |
|  | **ICC (Idose4)** | **95% CI** | **ICC (Precise)** | **95% CI** |
| Visualization | 0.797 | 0.447 - 1.22 | 0.854 | 0.643 - 1.08 |
| Critical reproduction | 0.902 | 0.755 - 1.06 | 0.878 | 0.707 - 1.05 |
| Visualization of large vessels | 0.963 | 0.889 -1.04 | 0.944 | 0.830 - 1.06 |
| Image Contrast | 0.951 | 0.849 - 1.06 | 0.878 | 0.707 - 1.05 |
| Image noise | 0.956 | 0.862 - 1.05 | 0.945 | 0.829 - 1.07 |

**Table 1b:** ICC-based interobserver agreement for qualitative image quality parameters

| **Qualitative parameters** | **Arterial** | | | |
| --- | --- | --- | --- | --- |
|  | **ICC (Idose4)** | **95% CI** | **ICC (Precise)** | **95% CI** |
| Visualization | 0.957 | 0.861 - 1.06 | 0.913 | 0.732 - 1.10 |
| Critical reproduction | 0.890 | 0.725 - 1.07 | 0.736 | 0.479 - 1.01 |
| Visualization of large vessels | 0.964 | 0.897 - 1.03 | 0.923 | 0.750 - 1.10 |
| Image Contrast | 0.913 | 0.783 - 1.06 | 0.936 | 0.811 - 1.07 |
| Image noise | 0.881 | 0.749 - 1.03 | 0.890 | 0.741 - 1.07 |

**Table 1c:** ICC-based interobserver agreement for qualitative image quality parameters

| **Qualitative parameters** | **Portal venous** | | | |
| --- | --- | --- | --- | --- |
|  | **ICC (Idose4)** | **95% CI** | **ICC (Precise)** | **95% CI** |
| Visualization | 0.923 | 0.725 - 1.15 | 0.923 | 0.740 - 1.12 |
| Critical reproduction | 0.804 | 0.576 - 1.06 | 0.814 | 0.538 - 1.11 |
| Visualization of large vessels | 0.876 | 0.753 - 0.995 | 0.886 | 0.725 - 1.05 |
| Image Contrast | 0.702 | 0.483 - 0.961 | 0.867 | 0.679 - 1.07 |
| Image noise | 0.808 | 0.587 - 1.05 | 0.925 | 0.794 - 1.07 |
